# Vitamin D3 promotes gastric cancer cell autophagy by mediating p53/AMPK/mTOR signaling

**DOI:** 10.3389/fphar.2023.1338260

**Published:** 2024-01-08

**Authors:** Yanan Wang, Qingmin He, Kang Rong, Mingyang Zhu, Xiaoxiao Zhao, Pengyuan Zheng, Yang Mi

**Affiliations:** ^1^ Henan Key Laboratory of Helicobacter Pylori and Microbiota and Gastrointestinal Cancer, Marshall B. J. Medical Research Center of Zhengzhou University, The Fifth Affiliated Hospital of Zhengzhou University, Zhengzhou, Henan, China; ^2^ Academy of Medical Science, Zhengzhou University, Zhengzhou, Henan, China; ^3^ Department of Gastroenterology, Ankang Central Hospital, Ankang, Shaanxi, China; ^4^ Department of Gastroenterology, The Fifth Affiliated Hospital of Zhengzhou University, Zhengzhou, Henan, China; ^5^ Department of Gastroenterology, The Second Affiliated Hospital of Zhengzhou University, Zhengzhou, Henan, China

**Keywords:** network pharmacology, molecular docking, vitamin D3, gastric cancer, molecular mechanism, autophagy

## Abstract

**Objective:** Vitamin D3 has the general properties of a lipid-soluble vitamin, but is also an active steroid hormone that can regulate the proliferation, apoptosis and differentiation of many tumor cells, and exerts anticancer activity against numerous malignancies. However, the mechanism underlying the effects of vitamin D3 on tumors is not fully understood. Here, we used network pharmacology and *in vitro* experimental approaches to explore the mechanism of vitamin D3 activity in the context of gastric cancer.

**Methods:** The Targetnet, SuperPred, SwissTargetPrediction, and PharmMapper databases were screened for potential drug-related targets, while we used data from the PharmGKB, Drugbank, OMIM, DisGeNET, CTD, and GeneCards databases to identify potential targets associated with gastric cancer. Disease-drug crossover genes were obtained by constructing Venn diagrams. Gene ontology and Kyoto Encyclopedia of Genomes (KEGG) enrichment analyses of crossover genes were conducted and STRING was used to generate protein interaction networks and identify core targets. CCK-8 experiments were performed and apoptosis detected to assess the effect of vitamin D3 on gastric cancer cells. Western blotting was applied to detect p53/AMPK/mTOR signaling, as well as autophagy-, cell cycle-, and apoptosis-related proteins.

**Results:** A total of 485 targets of vitamin D3 activity were obtained and 1200 gastric cancer disease-related targets discovered. Further, 60 potential targets for vitamin D3 in gastric cancer treatment were identified. KEGG analysis indicated that potential targets were mainly involved in the cell cycle, HIF-1 signaling, and the AMPK pathway, among other pathways. These findings were validated using cellular experiments, which demonstrated that the viability of AGS and SGC-7901 cells was impeded by vitamin D3. Further, vitamin D3 promoted apoptosis and inhibited the cell cycle in those cell lines, as well as activating the p53/AMPK/mTOR pathway, which promotes autophagy and inhibits tumor development.

**Conclusion:** Our network pharmacological analyses provide preliminarily data supporting a role for vitamin D3 in promoting autophagy and apoptosis in gastric cancer cells, and in activating the p53/AMPK/mTOR pathway, which inhibits gastric cancer cell proliferation. Our findings demonstrate the molecular mechanism underlying the effect of vitamin D3 in cure of gastric cancer.

## 1 Introduction

Gastric cancer (GC) is the fifth most malignant and fourth most common tumor resulting in cancer-related death (Siegel et al., 2021; Sung et al., 2021). Notably, GC-associated morbidity has been rising steadily in young adults (<50 years of age) in recent years. Besides *Helicobacter pylori* infection, GC is also associated with other factors, including lifestyle features, such as alcohol consumption and smoking (Tramacere et al., 2012; Tan and Yeoh, 2015; Lu et al., 2021; Lordick et al., 2022). Mean age at time of GC diagnosis is 65 years and comorbidities are common. Various symptoms are associated with GC, including dysphagia, dyspepsia, and vomiting, depending on the location of the tumor (Lordick et al., 2022). Currently, surgical resection, radiotherapy, immunotherapy, and molecularly targeted drugs are common therapeutic modalities for GC, while the most effective treatment is chemotherapy (Song et al., 2017). The prognosis is poor (Miller et al., 2019) and identification of curative treatments for GC, by finding effective drugs, is crucial.

Vitamin D3 is a well-known essential hormone that is involved in regulating the absorption of phosphate and calcium to fully mineralize the skeletal system (Gao et al., 2018). Vitamin D3 deficiency is linked to a number of diseases, including high blood pressure, cardiovascular disease, falls, diabetes, and cancer (Mahendra et al., 2018). Vitamin D3 has several unique roles, including acting as an antioxidant or immunomodulator. Further, as it can cross cell membranes, vitamin D3 performs multiple functions within cells, including involvement in gene regulation. Further, vitamin D3 regulates the proliferation, invasion (Shah et al., 2021), differentiation, and apoptosis of cancer cells, including GC cells. Moreover, the involvement of vitamin D3 in angiogenesis and cellular molecular signaling suggest that it may be associated with cancer incidence, prognosis, and mortality (Fleet, 2008; Fleet et al., 2012; Rosen et al., 2012). A study reported that increased serum levels of vitamin D3 may lower the risk of GC (Ren et al., 2012); however, the exact mechanism involved requires further investigation.

Using computational software, network pharmacology analysis can be applied to validate drug-actionable targets, by combining network biology and poly-pharmacology. Furthermore, we used network pharmacology to explore the potential therapeutic mechanisms of vitamin D3 (Hopkins, 2008). In addition to providing a way to understand drug side effects and toxicity, network pharmacology encourages drug development, and new approaches to disease diagnosis, definition, and treatment have been created using this method (Nogales et al., 2022). Relationships between diseases and drugs have been established through exploring the interactions of proteins and diseases. Here, we applied network and molecular pharmacology to investigate the molecular mechanisms underlying the effect of vitamin D3 in the context of GC. Further, the anti-tumor effects of vitamin D3 were verified in cell culture experiments.

## 2 Materials and methods

### 2.1 Network pharmacology

#### 2.1.1 Vitamin D3 target prediction

**FIGURE 1:**
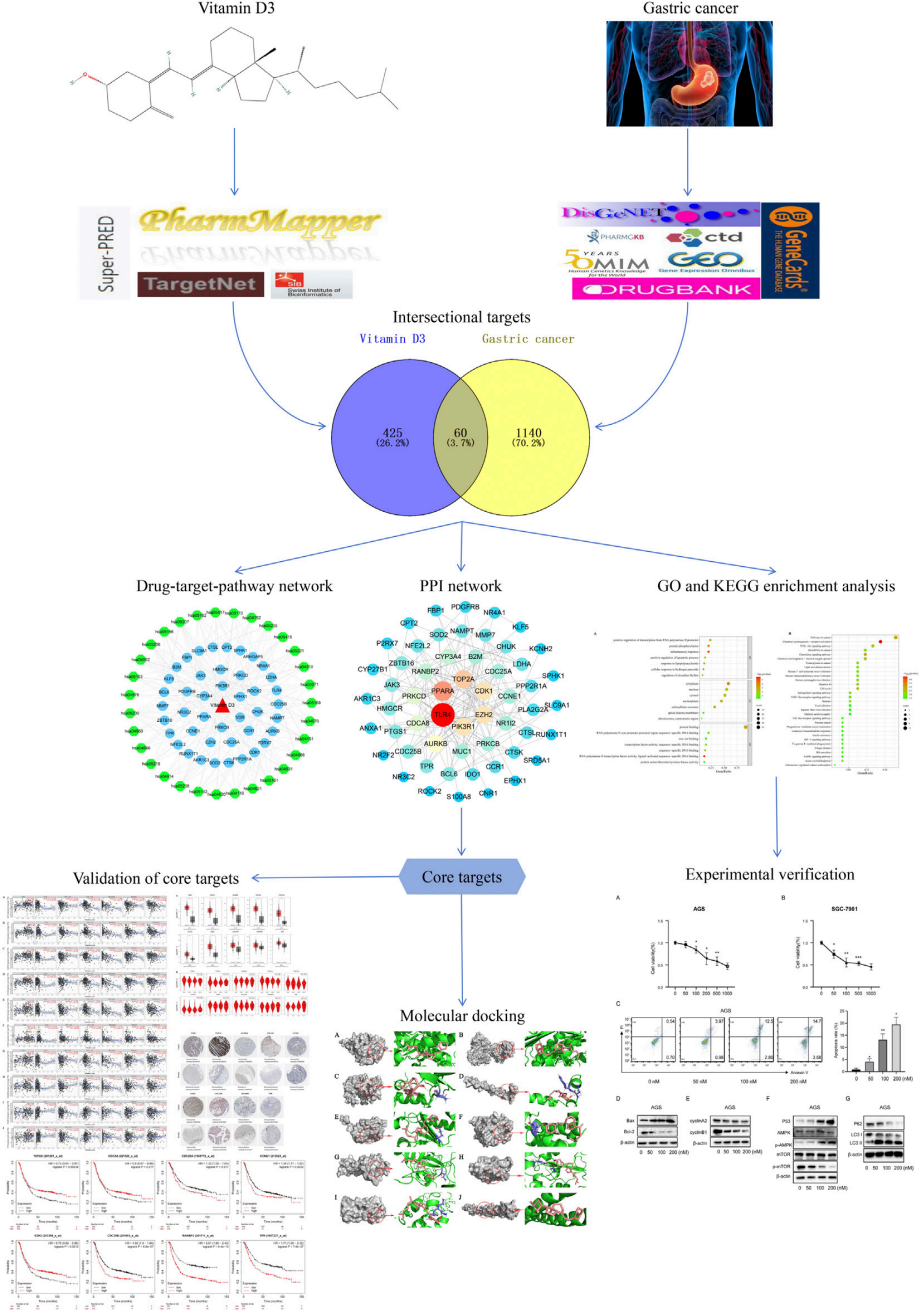
A detailed illustration of this study.

The databases used are listed in Table 1. Aworkflow diagram of the analysis is shown in Figure 1. To obtain characterization information and physicochemical parameters for vitamin D3, PubChem (Kim et al., 2021) was searched using the search term, CAS: 67–97-0. Target prediction was conducted using the Pharmmapper (Wang et al., 2017), TargetNet (Yao et al., 2016), SwissTargetPrediction (Daina et al., 2019), and Superpred (Nickel et al., 2014) websites, using SDF files of vitamin D3 or Canonical SMILES for prediction. Targets were converted to gene names using the UniProt database (Pundir et al., 2017). Finally, obtained target genes were merged, duplicates removed, and the results considered targets of vitamin D3.

**TABLE 1:** Information on the database used to screen for gastric cancer with vitamin D3.

Name	URL
PubChem	https://pubchem.ncbi.nlm.nih.gov/
PharmMapper	http://lilab-ecust.cn/pharmmapper/
SuperPred	https://prediction.charite.de/
Targetnet	http://targetnet.scbdd.com/
SwissTargetPrediction	http://www.swisstargetprediction.ch/
GEO	https://www.ncbi.nlm.nih.gov/geo/
OMIM	https://www.omim.org/
GeneCards	https://www.genecards.org/
DisGeNET	https://www.disgenet.org/
GEPIA	http://gepia.cancer-pku.cn/
HPA	https://www.proteinatlas.org/
TIMER	https://cistrome.shinyapps.io/timer/
Uniprot	https://www.uniprot.org/
STRING	https://cn.string-db.org/
RCSB PDB	https://www.rcsb.org/
Bioinformatics	http://www.bioinformatics.com.cn/
DAVID	https://david.ncifcrf.gov/
Venny 2.1.0	https://bioinfogp.cnb.csic.es/tools/venny/index.html
KEGG Mapper	https://www.kegg.jp/kegg/mapper/
Drugbank	https://go.drugbank.com/
CTD	https://ctdbase.org
Kaplan-Meier plotter	https://kmplot.com/
Cytoscape	https://cytoscape.org/
PharmGKB	https://www.pharmgkb.org/
DisGeNET	https://www.disgenet.org/

#### 2.1.2 Potential GC target identification

The GSE29998, GSE54129, GSE19826, GSE81948, and GSE118916 datasets were selected from the GEO database (Clough and Barrett, 2016). Genes satisfying GEO2R: |log fold-change (FC)| >1, adjusted *p*-value < 0.05 were designated as differentially expressed. Differentially expressed genes were illustrated using Volcano plots generated by bioinformatics website, following de-duplication and merging of those retrieved from the five GEO datasets. The Drugbank (Wishart et al., 2018), PharmGKB (Barbarino et al., 2018), GeneCards (Stelzer et al., 2016), OMIM (Amberger et al., 2015), DisGeNET (Piñero et al., 2021), and CTD (Davis et al., 2023) databases were used to obtain GC targets. Results from the six databases were merged after removal of duplicates. Finally, data from the five GEO databases were intersected with those from these six databases, then duplicates removed to obtain GC-related targets.

#### 2.1.3 Common drug-disease targets and PPI network construction

To identify common targets, vitamin D3 and GC targets were intersected using Venny 2.1.0 software and a Venn diagram generated. Those genes obtained represented potential targets of vitamin D3 involved in its effects on GC. We used the STRING database (Szklarczyk et al., 2023) to obtain protein-protein interaction (PPI) networks (Ding and Kihara, 2019) for identified targets, and the downloaded tsv file imported into Cytoscape 3.7.2 software (Shannon et al., 2003) for visualization. The “cytohubba” plugin was used to generate the PPI network and Maximal Clique Centrality (MCC) selected to calculate the top 10 hub targets.

#### 2.1.4 Gene ontology (GO) and kyoto encyclopedia of genes and genomes (KEGG) enrichment analyses

GO and KEGG enrichment analyses were performed using DAVID (Huang da et al., 2009; Sherman et al., 2022) to identify potential targets of vitamin D3 in GC. The data were collated and visualized by bioinformatics analysis and displayed as bubble maps. Vitamin D3, KEGG pathways, and potential targets of vitamin D3 were subjected to drug-target-pathway network analysis using Cytoscape 3.7.2. Vitamin D3, genes, or pathways were represented by nodes, and the roles of biomolecules in relation to each other were represented by connecting lines.

#### 2.1.5 Molecular docking

Vitamin D3 was downloaded from PubChem in SDF format (Kim et al., 2023) and converted to mol2 format using OpenBabel-3.1.1 (O'Boyle et al., 2011). Vitamin D3 was twisted in AutoDockTools 1.5.7 (Morris et al., 2009) and exported as pdbqt format files. Details of all targets got from the PDB database are presented in Table 2 in PDB format. Proteins were de-hydrated and ligands removed in PyMOL (Seeliger and de Groot, 2010), and hydrogenated using AutoDockTools 1.5.7. Results are displayed in pdbqt format. We imported the pdbqt files of proteins and vitamin D3 into AutoDockTools 1.5.7. To construct the docking box, the protein was placed in the center and the docking box sized to cover the protein, with vitamin D3 outside of the box. The parameter settings are presented in Table 3. AutoDockTools 1.5.7 was used for docking. The likelihood of a protein binding to vitamin D3 was judged according to the magnitude of the binding energy. Vitamin D3 and protein pairs with lower binding energies had higher affinity and, consequently, their conformations were more stable. We used the PyMOL to visualize the molecular docking results based on binding energy values.

**TABLE 2:** Protein target details in the PDB database using method of X-RAY DIFFRACTION.

Targets	PDB ID	Resolution(Å)	R-Value free	R-Value work	R-Value Observed
CDK1	6GU6	2.330	0.246	0.201	0.203
TOP2A	4R1F	2.510	0.243	0.208	0.211
AURKB	4AF3	2.750	0.264	0.205	0.208
CDCA8	2RAW	2.400	0.298	0.248	0.254
CDC25A	1C25	2.300	0.296	0.227	0.227
CCNE1	8H6P	2.440	0.292	0.221	0.222
EZH2	4M15	1.520	0.186	0.172	0.172
CDC25B	1YMD	1.700	0.209	0.188	0.190
RANBP2	7MNK	1.100	0.167	0.155	0.155
TPR	5TO5	2.500	0.282	0.229	0.232

**TABLE 3:** Data generated in molecular docking between vitamin D3 and hub genes.

Target name	PDB ID	Spacing (angstrom)	Center Grid Box			Binding energy (kcal/Mol)
			X center	Y center	Z center	
CDK1	6GU6	0.569	18.379	16.239	9.876	−6.590
TOP2A	4R1F	1.000	40.855	24.047	42.126	−5.000
AURKB	4AF3	0.492	16.434	−17.261	−1.316	−8.790
CDCA8	2RAW	0.608	14.678	−4.981	7.710	−6.470
CDC25A	1C25	0.497	6.103	31.844	73.111	−7.170
CCNE1	8H6P	0.464	28.866	−9.138	−22.54	−7.610
EZH2	4M15	0.519	28.033	21.725	10.468	−6.930
CDC25B	1YMD	0.447	19.636	11.507	25.266	−6.980
RANBP2	7MNK	0.442	40.851	19.138	7.535	−9.410
TPR	5TO5	1.000	−3.344	−4.979	45.486	−3.650

#### 2.1.6 External validation of hub genes

Pathological staging and gene expression of hub genes were verified in GEPIA (Tang et al., 2017), with the following parameters: *p*-value Cutoff is 0.01and |Log2FC| Cutoff is 1. Level of hub genes in GC tissue and comparisons of the protein level of hub genes in normal and gastric cancer tissues were tested using The Human Protein Atlas database (Uhlén et al., 2015). We used the TIMER database (Li et al., 2017) to test the association of hub genes with degree of immune infiltration cells. Data from Kaplan-Meier Plotter (Győrffy, 2023), an online database that enables online survival analysis of patients with a wide range of tumors, were analyzed to assess the value of hub targets as prognostic indicators.

### 2.2 Experimental verification

#### 2.2.1 Cell viability assay

The AGS and GSC-7901 cells were inoculated into 96-well plates (5000 cells/well) and cultured for 24 h. Then, vitamin D3 (0–1000 nM) was used and cells were incubated for a further 24 h. Then we added the CCK-8 (10 μL/well; Meilunbio, MA0218-1) and incubation at 37°C for 1 h. We measured the optical density at 450 nm.

#### 2.2.2 Cell apoptosis assay

AGS and GSC-7901 cells were inoculated into 24-well plates, then following adhesion of the cells to the plates, vitamin D3 was added at the indicated concentration. 24 h later, cells were digested using EDTA-free trypsin. we stained them using an Annexin V-FITC/propidium iodide (PI) staining kit (KeyGene BioTech), and apoptosis levels were detected using flow cytometry.

#### 2.2.3 Western blotting

AGS and SGC-7901 GC gastric cells were incubated with the different concentrations of vitamin D3 for 24 h. Then total protein samples were extracted using RIPA lysate buffer, supplemented with PMSF (1×) and PIC (1×), then added loading buffer, samples heated at 100°C about 10 min, and total proteins were separated by SDS-PAGE. Then proteins were transferred to the PVDF membranes. 5% skimmed milk powder was used to block the membranes. We incubated the membranes at 4°C almost 12 h in the following primary antibodies: anti-P62/SQSTM1 (Boster Biological Technology, PB0458), AMPK alpha polyclonal (Proteintech, 10929-2-AP), anti-phospho-AMPK alpha 1 (Ser356) polyclonal (Bioss, bs-14318R), mTOR monoclonal (Proteintech, 66888-1-Ig), anti-mTOR (phospho S2448) (Abcam, ab109268), p53 monoclonal (Proteintech, 60283-2-Ig), LC3 polyclonal (Proteintech, 14600-1-AP), BAX polyclonal (Proteintech, 50599-2-Ig), Bcl2 polyclonal (Proteintech, 26593-1-AP), Cyclin A2 polyclonal (Proteintech, 18202-1-AP), Cyclin B1 polyclonal (Proteintech, 55004-1-AP), and β-actin (Abcam, ab6276). Membranes were then incubated with anti-mouse/rabbit IgG secondary antibody for 1 h after being washed in TBST for 5 min 3 times once and washed a further 3 times for 10 min each in TBST before exposure using an exposure machine (BIO-RAD). All experiments were repeated three times.

### 2.3 Statistical analysis

GraphPad Prism 8 software was used to analyze for each experiment which is a total of three independent samples. Data are expressed as mean ± SD. The *t*-test was used to analyze statistical differences between two groups. A significant difference was mean *p* < 0.05. We performed the statistical analysis of data using GraphPad Prism version 8.0 (Berkman et al., 2019).

## 3 Results

### 3.1 Network pharmacology analysis

#### 3.1.1 Identification of vitamin D3 and GC target genes

**FIGURE 2:**
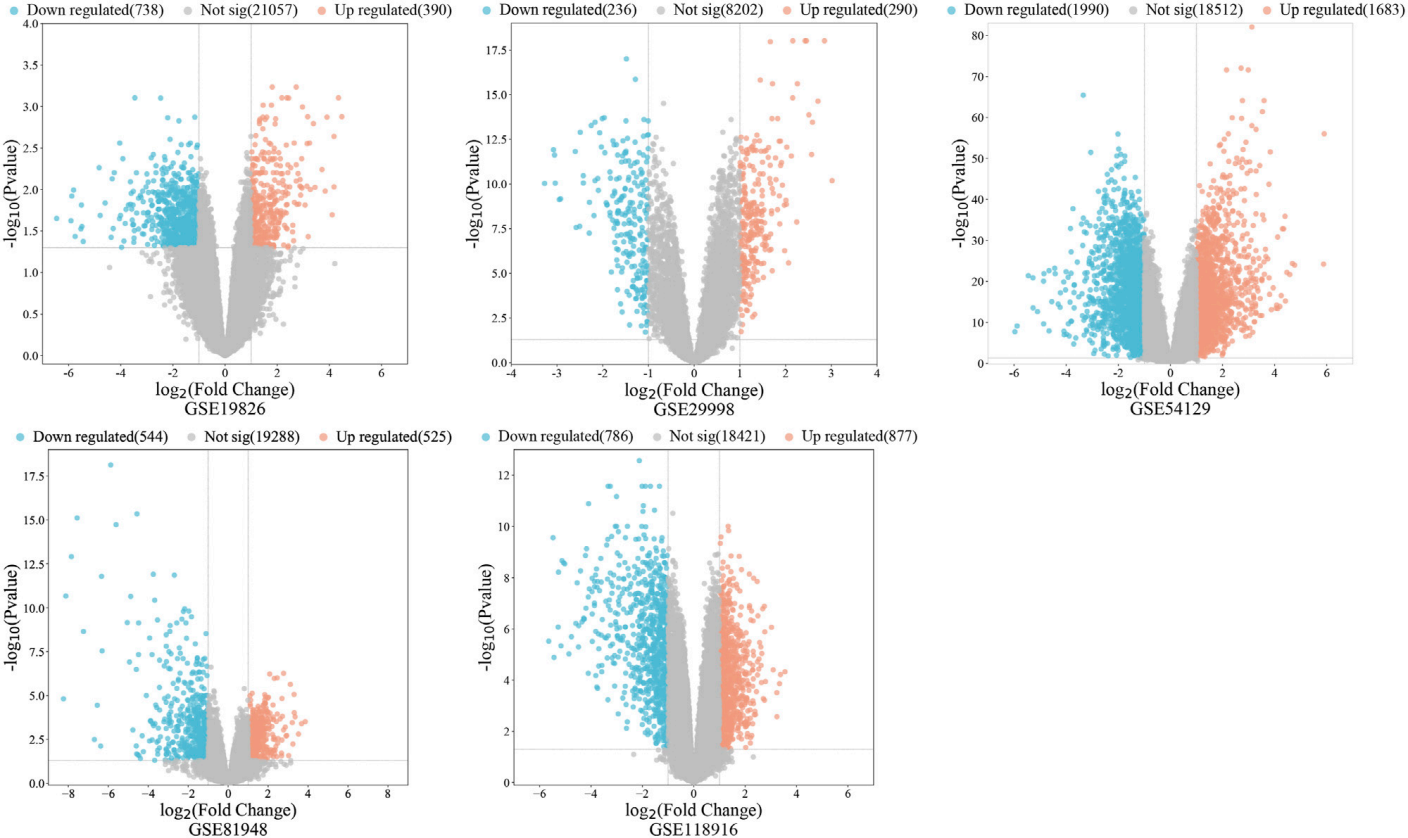
The volcano diagram represents gastric cancer associated DEGs.

A total of 485 vitamin D3 targets were obtained and 5455 differentially expressed genes were screened from the GSE19826, GSE29998, GSE54129, GSE81948, GSE 118916 datasets (Figure 2). Further, 3394 GC targets were acquired through screening of Drugbank, PharmGKB, GeneCards, OMIM, DisGeNET, and CTD. By intersecting the 3394 GC target genes and 5455 differentially expressed genes, we obtained 1200 GC-related targets.

#### 3.1.2 Identification of common targets and PPI network construction

**FIGURE 3:**
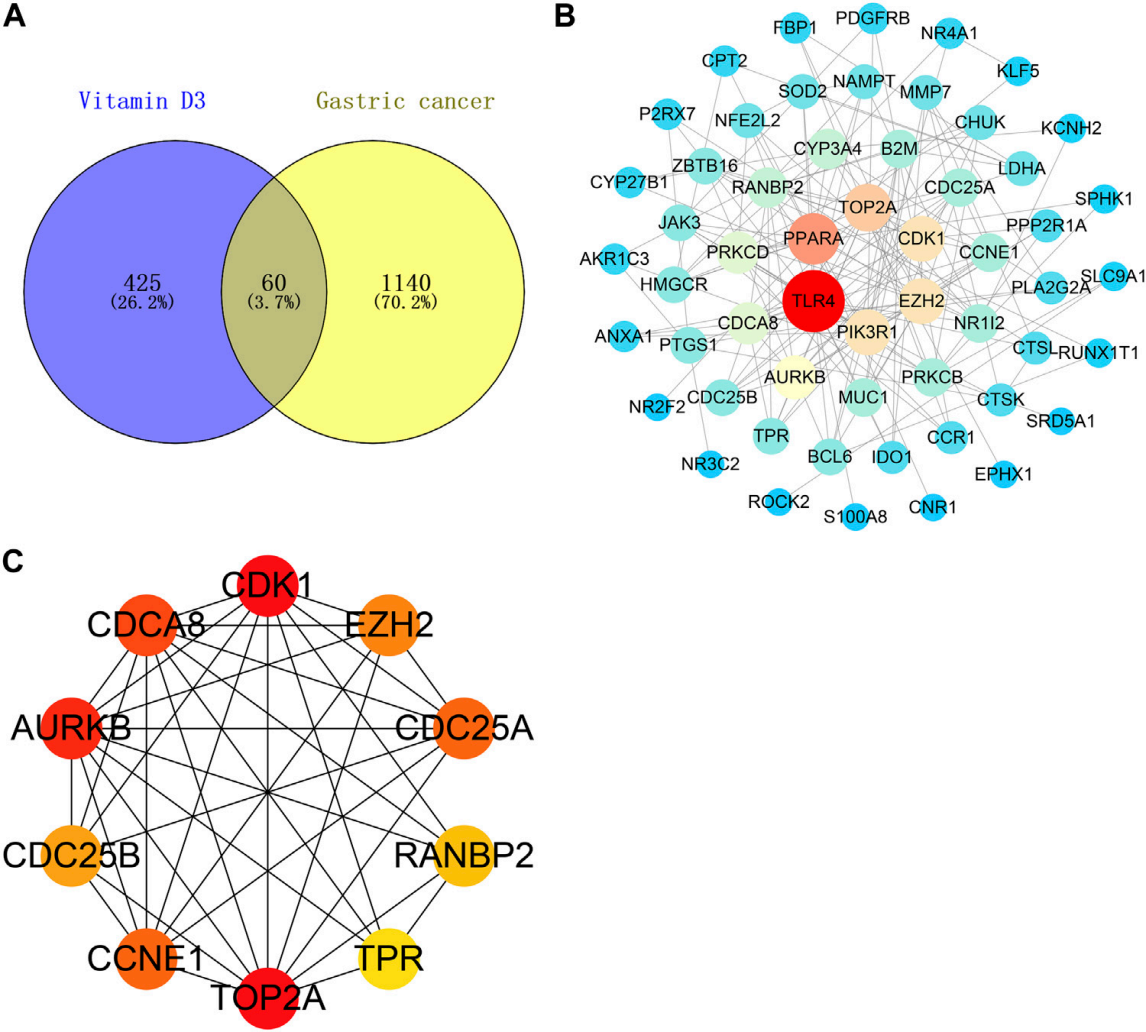
Vitamin D3 acts on relevant targets in gastric cancer and PPI network. **(A)** Venn diagram indicating cross genes for vitamin D3 and gastric cancer. **(B)** PPI networks indicate potential targets for vitamin D3 action on gastric cancer. **(C)** Hub genes.

A Venn diagram was created by intersecting the 485 vitamin D3 targets with the 1200 GC targets using Venny 2.1.0; we identified 60 potential targets for the effects of vitamin D3 in GC (Figure 3A) and constructed a PPI network of these using STRING. The tsv file was used in Cytoscape 3.7.2 and, after removing isolated targets, a final PPI network containing 56 nodes and 151 edges was generated (Figure 3B). “Network analyzer” was used to analyze the degree values of targets, which were represented in the PPI network as variation in node size and color depth. PPI network maps of potential targets were then analyzed using CytoHubba, which identified the following 10 core targets: CDK1, TOP2A, AURKB, CDCA8, CDC25A, CCNE1, EZH2, CDC25B, RANBP2, and TPR (Figure 3C).

#### 3.1.3 GO and KEGG enrichment analyses and drug-target-pathway network construction

**FIGURE 4:**
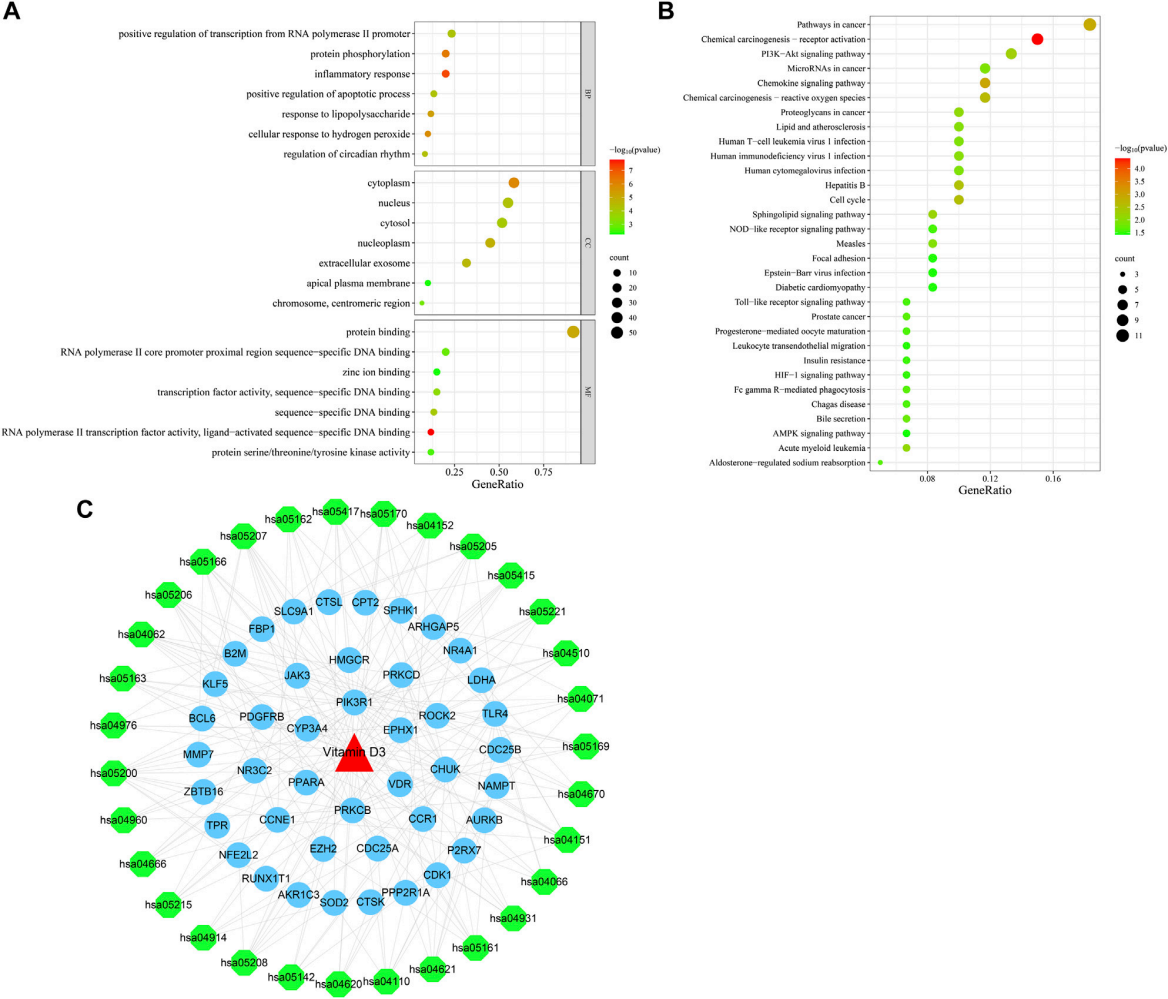
Bubble plots of enrichment analysis. **(A)** GO functional enrichment analysis. **(B)** KEGG pathway enrichment analysis. **(C)** Drug-target-pathway network diagram.

Next, we conducted GO function enrichment analysis of the 60 intersection targets, resulting in a total of 189 entries (Figure 4A), including 129, 34 and 26 in the Biological Progress (BP), Molecular Function categories (MF), and Cellular Component (CC) respectively. Bubble maps of the top 7 enriched terms for BP, MF, and CC were then generated. Intersection targets were enriched for the BP terms: positive regulation of transcription from RNA polymerase II promoter, protein phosphorylation, inflammatory response, and positive regulation of apoptotic process, among others. MF enrichment terms were mainly involved in RNA polymerase II core promoter proximal region sequence-specific DNA binding, transcription factor activity, and protein binding. Additionally, enriched CC terms were primarily associated with the cytoplasm and nucleus.

KEGG pathway enrichment analysis identified 49 enriched signaling pathways, then the top-31 were visualized as a bubble map (Figure 4B). Our results suggested that vitamin D3 is involved in cancer-related pathways, including chemical carcinogenesis-receptor activation, AMPK signaling, MicroRNAs in cancer, and PI3K-Akt signaling pathway among others. Next, the top-31 pathways were used in Cytoscape 3.7.2 to draw a drug-target-pathway network map for vitamin D3 treatment effects in GC (Figure 4C). Green represents pathways, blue represents targets, and red represents vitamin D3. The results showed that vitamin D3 can affect multiple targets and signaling pathways related to GC.

#### 3.1.4 Molecular docking analysis validates interactions of vitamin D3 and hub genes

**FIGURE 5:**
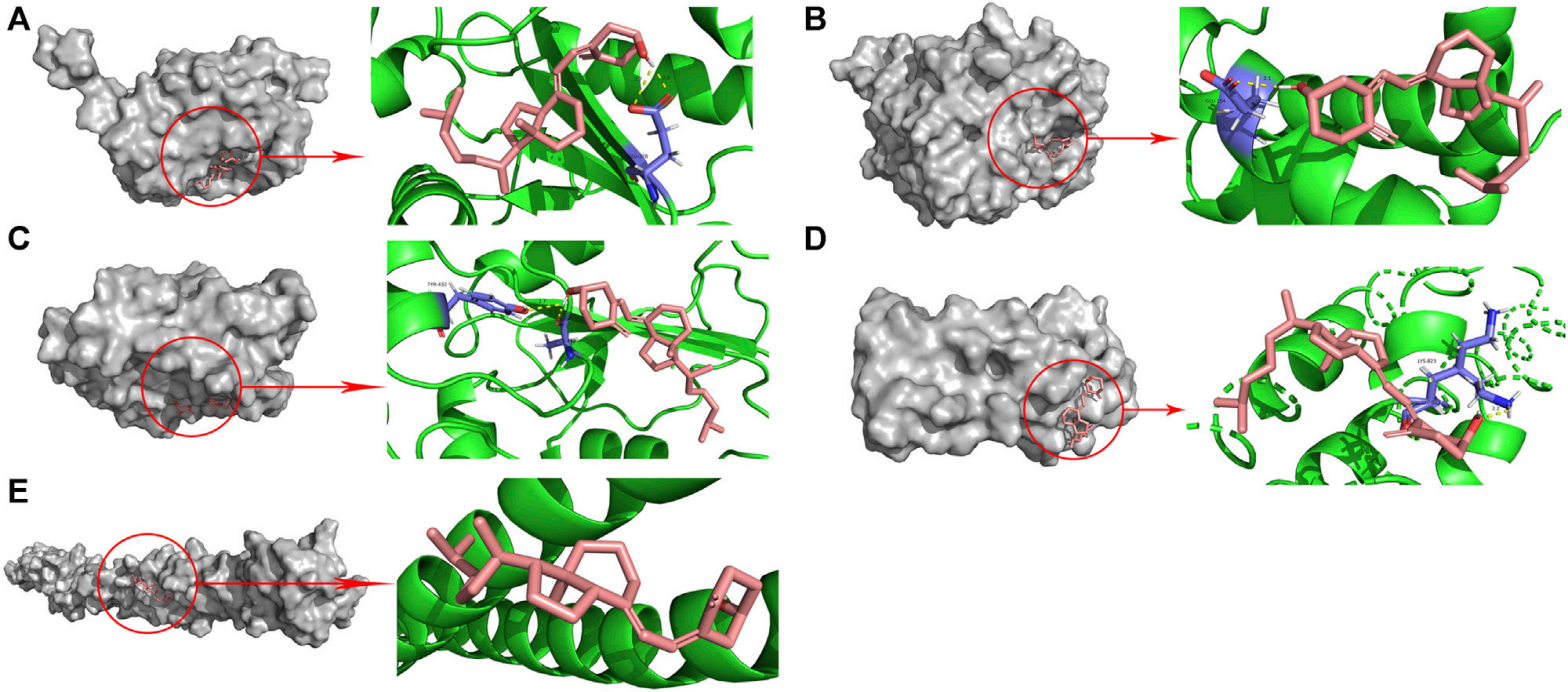
Molecular docking pattern of vitamin D3 with 5 hub target proteins. **(A)**. CDC25A **(B)**. CCNE1 **(C)**. CDC25B **(D)**. RANBP2 **(E)**. TPR.

To assess whether vitamin D3 can stably bind to target proteins identified by network analysis under physiological conditions, we conducted molecular docking analysis of vitamin D3 with its hub genes. Molecular docking between the 10 hub target proteins and vitamin D3 was visualized (Figure 5; Supplementary Figure S1) and the corresponding binding energies are collated in Table 3; the binding capacity of a ligand to a receptor is usually determined by the binding energy. Our analysis identified that the binding energies of vitamin D3 to the identified hub genes were ≤3.650 kcal/Mol, which suggests that vitamin D3 can readily bind to these targets, with relatively stable conformation (Li et al., 2021). These core targets are closely related to tumor development and may be tightly linked to the anticancer effects of vitamin D3.

### 3.2 Validation of hub genes

#### 3.2.1 Association of hub genes with survival of patients with GC

**FIGURE 6:**
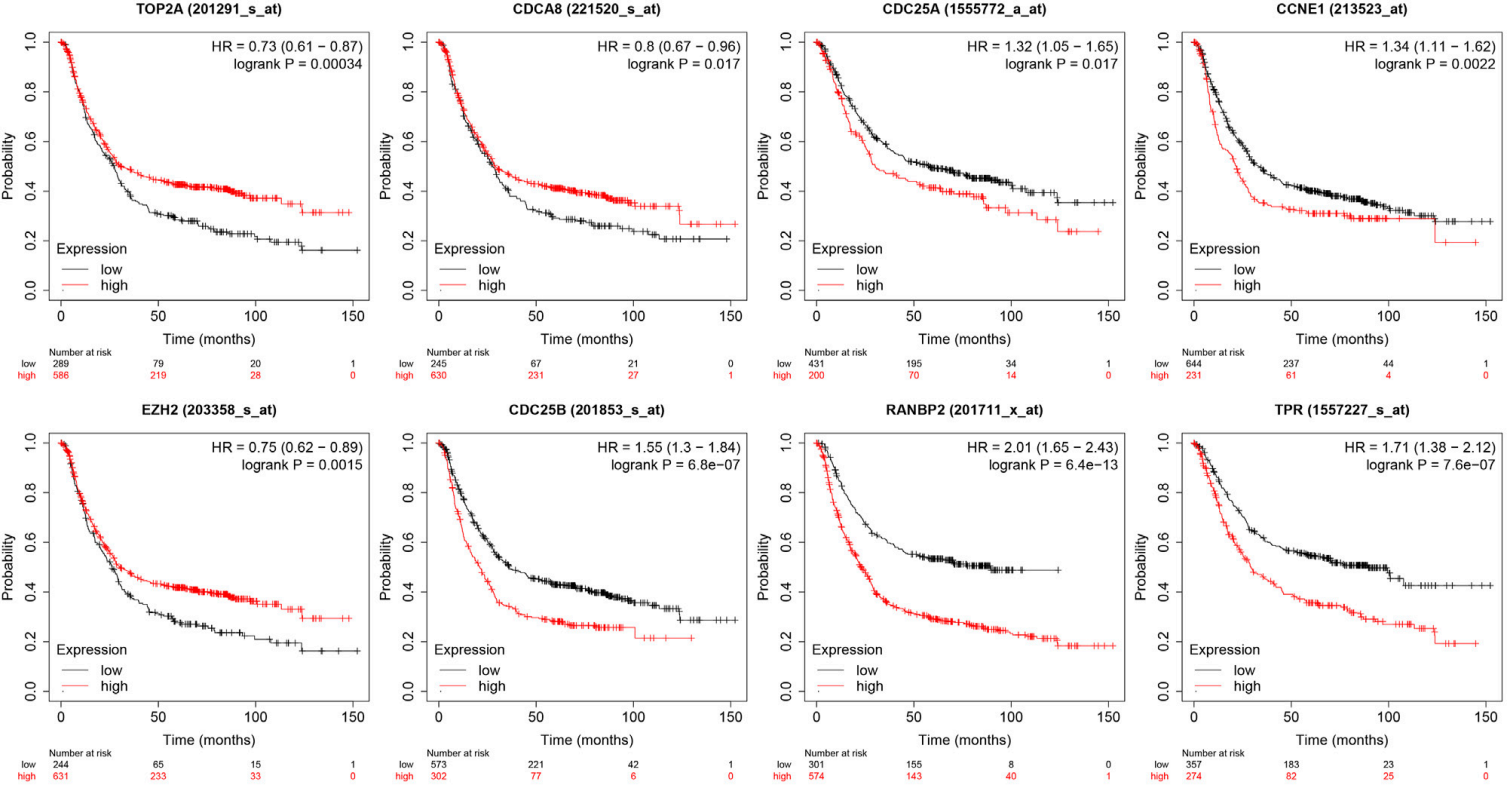
Relationship between 10 hub genes expression levels and survival prognosis.

Analysis of the relationships between core target gene levels and patient survival was conducted using the Kaplan-Meier Plotter database and demonstrated significant associations between RNABP2, CDC25B, CDC25A, CCNE1, and TPR levels with poor prognosis of patients with GC (*p* < 0.05) (Figure 6). Our next analysis mainly focused on these 5 hub genes. Cox proportional risk modelling was used to analyze the clinical significance of GC hub genes and immune infiltration cells. Our analyses showed significant associations with overall survival in age, stage 3, stage 4, macrophage, CDK1, EZH2, and AURKB in patients with GC (Table 4).

**TABLE 4:** Analysis of tumor infiltrating immune cells and hub genes using the Cox proportional hazards model.

	Coef	HR	95%CI_l	95%CI_u	*p*.value
Age	0.033	1.034	1.011	1.057	0.004
gendermale	0.309	1.362	0.862	2.149	0.185
raceBlack	0.599	1.820	0.725	4.569	0.203
raceWhite	0.187	1.206	0.689	2.109	0.512
stage2	0.784	2.190	0.948	5.058	0.066
stage3	1.027	2.792	1.293	6.031	0.009
stage4	1.403	4.068	1.336	12.385	0.014
Purity	−0.616	0.540	0.229	1.276	0.160
B_cell	3.526	34.000	0.189	6129.535	0.183
CD8_Tcell	0.570	1.768	0.055	56.818	0.747
CD4_Tcell	−2.946	0.053	0.000	17.706	0.321
Macrophage	7.031	1130.630	15.563	82136.112	0.001
Neutrophil	−1.762	0.172	0.000	758.472	0.681
Dendritic	−0.163	0.850	0.038	19.121	0.918
CDK1	0.500	1.649	1.054	2.578	0.029
TOP2A	−0.133	0.876	0.623	1.231	0.446
AURKB	0.467	1.595	1.016	2.502	0.042
CDCA8	−0.350	0.705	0.443	1.121	0.139
CDC25A	−0.169	0.845	0.545	1.308	0.449
CCNE1	0.095	1.100	0.922	1.312	0.289
EZH2	−0.543	0.581	0.348	0.968	0.037
CDC25B	−0.137	0.872	0.660	1.152	0.334
RANBP2	0.063	1.066	0.574	1.977	0.840
TPR	0.323	1.381	0.720	2.648	0.331

#### 3.2.2 Hub genes mRNA expression levels

**FIGURE 7:**
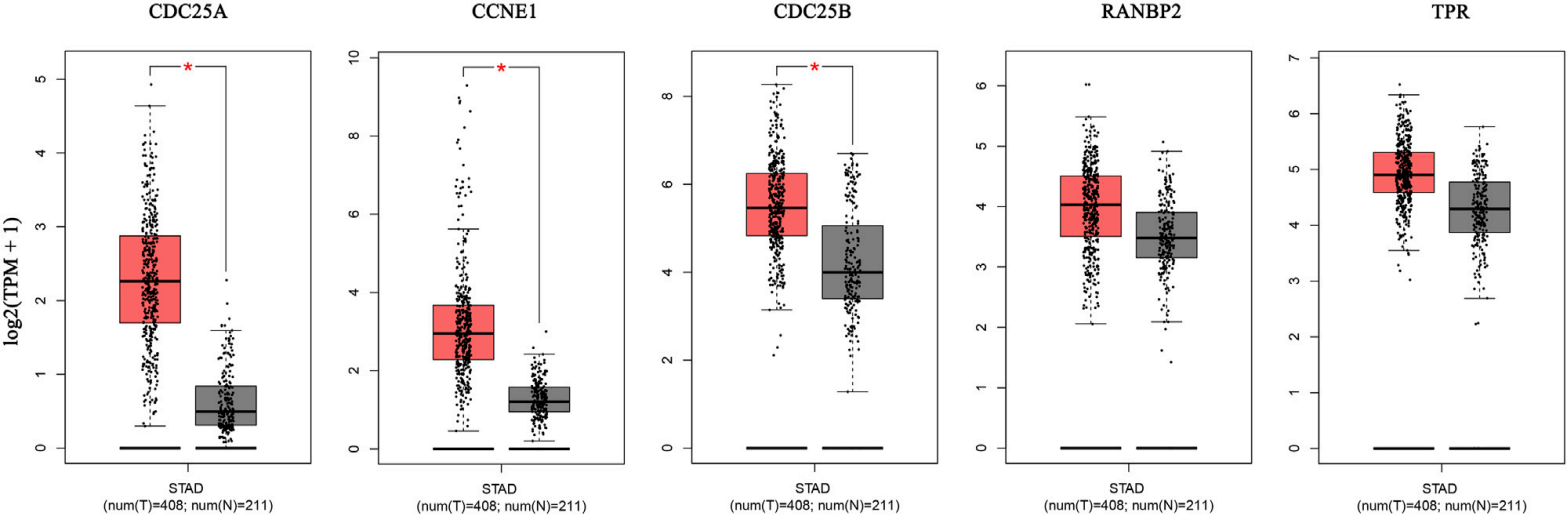
The mRNA expression of 5 hub genes in tumor tissue (red) and normal tissue (grey) in the GEPIA database.

The GEPIA database was used to validate the differences in level of core targets between gastric cancer and normal tissues. Significantly higher mRNA expression of 3 core targets, *CDC25A, CCNE1, CDC25B,* were detected in GC than in normal tissue samples. Further, levels of the remaining two genes, *TPR* and *RNABP2,* were higher in gastric cancer tissue, but the differences were not significant (*p* > 0.01) (Figure 7). And other 5 genes, *CDK1, TOP2A, AURKB, CDCA8, EZH2,* were significantly higher in GC tissue (Supplementary Figure S2). We next analyzed the mRNA levels of hub genes according to GC pathological stage; no significant differences among pathological stages were detected for any genes (*p* > 0.01) (Supplementary Figure S3).

#### 3.2.3 Protein expression levels of hub genes

**FIGURE 8:**
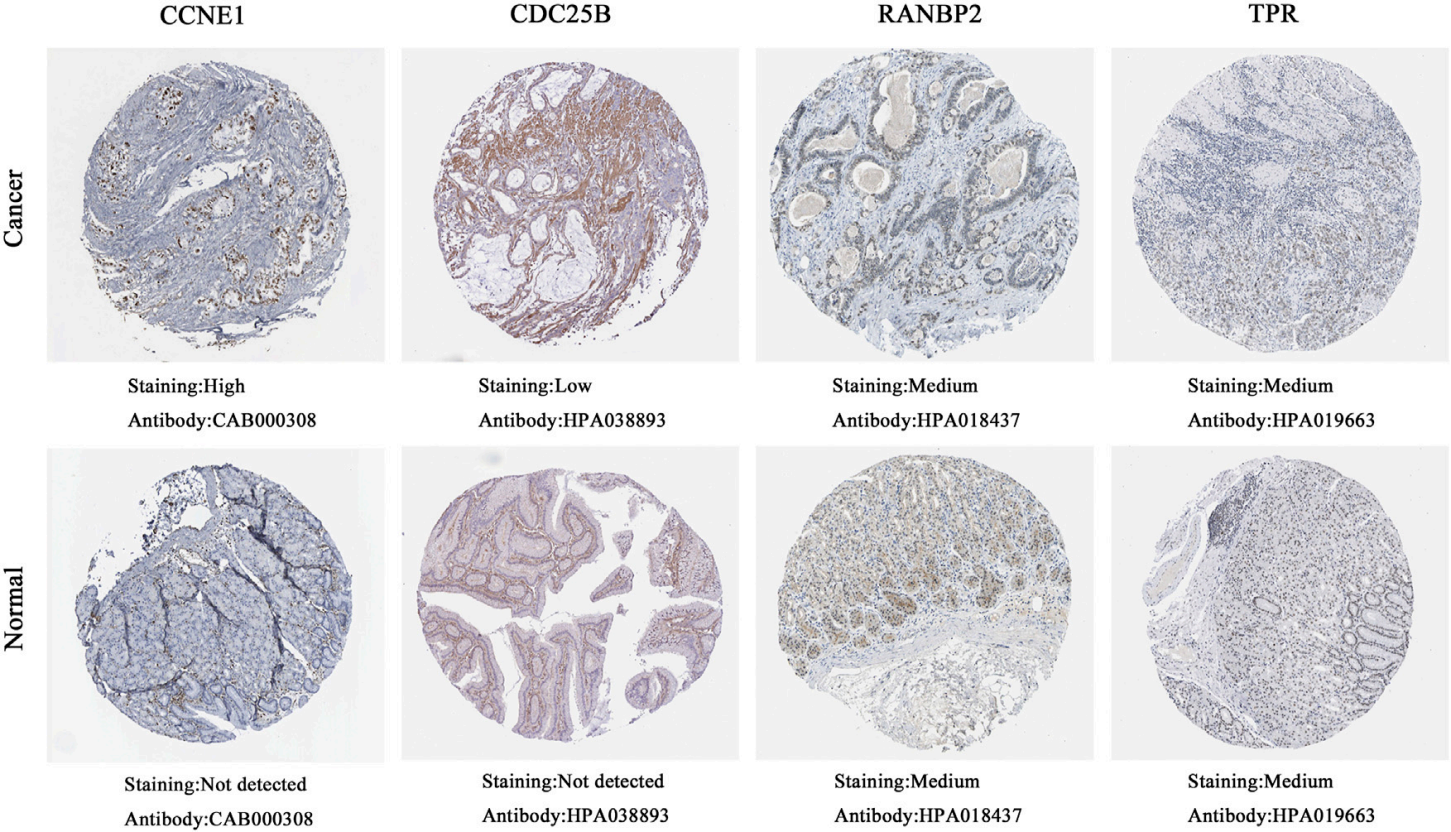
The protein expression of 4 hub genes in immunohistochemical images in the HPA database.

Next, we retrieved immunohistochemical images of hub genes from the HPA database and found that 2 core proteins, CCNE1 and CDC25B, were differentially expressed between normal tissues and GC. No significant differences in RANBP2 and TPR were detected between GC and normal tissues (Figure 8). The HPA database did not contain immunohistochemical data for CDC25A. And other proteins, CDK1, TOP2A, AURKB and CDCA8, were higher in GC tissue (Supplementary Figure S4).

#### 3.2.4 Association of hub genes with immune cell infiltration

**FIGURE 9:**
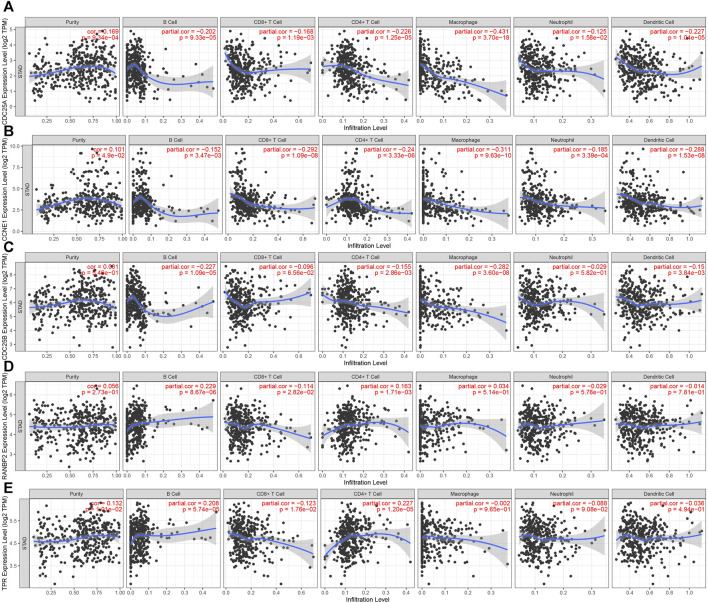
Correlation between 5 hub genes and immune cell infiltration. **(A)**. CDC25A **(B)**. CCNE1 **(C)**. CDC25B **(D)**. RANBP2 **(E)**. TPR.

Analysis of associations between the 5 hub genes and immune infiltration cells showed that all of the hub molecules, except CDC25B and RANBP2, were positively correlated with purity. CCNE1 and CDC25A levels were negatively correlated with B cell, neutrophil, CD8^+^ T cell, macrophage, CD4^+^ T cell and dendritic cell infiltration. TRP genes were negatively correlated with CD8^+^ T cell infiltration, and positively related with CD4^+^ T cell and B cell infiltration. CDC25B was negatively with B cell, macrophage, CD4^+^ T cell and dendritic cell infiltration. RANBP2 was positivity correlated with B cell, and CD4^+^ T cell infiltration and negatively correlated with CD8^+^ T cells (Figure 9). Finally, AURKB and CDCA8 levels were negatively correlated with all analyzed immune cell types except neutrophils (Supplementary Figure S5).

### 3.3 *In vitro* experiments

#### 3.3.1 Vitamin D3 inhibits GC cell viability in vitro

**FIGURE 10:**
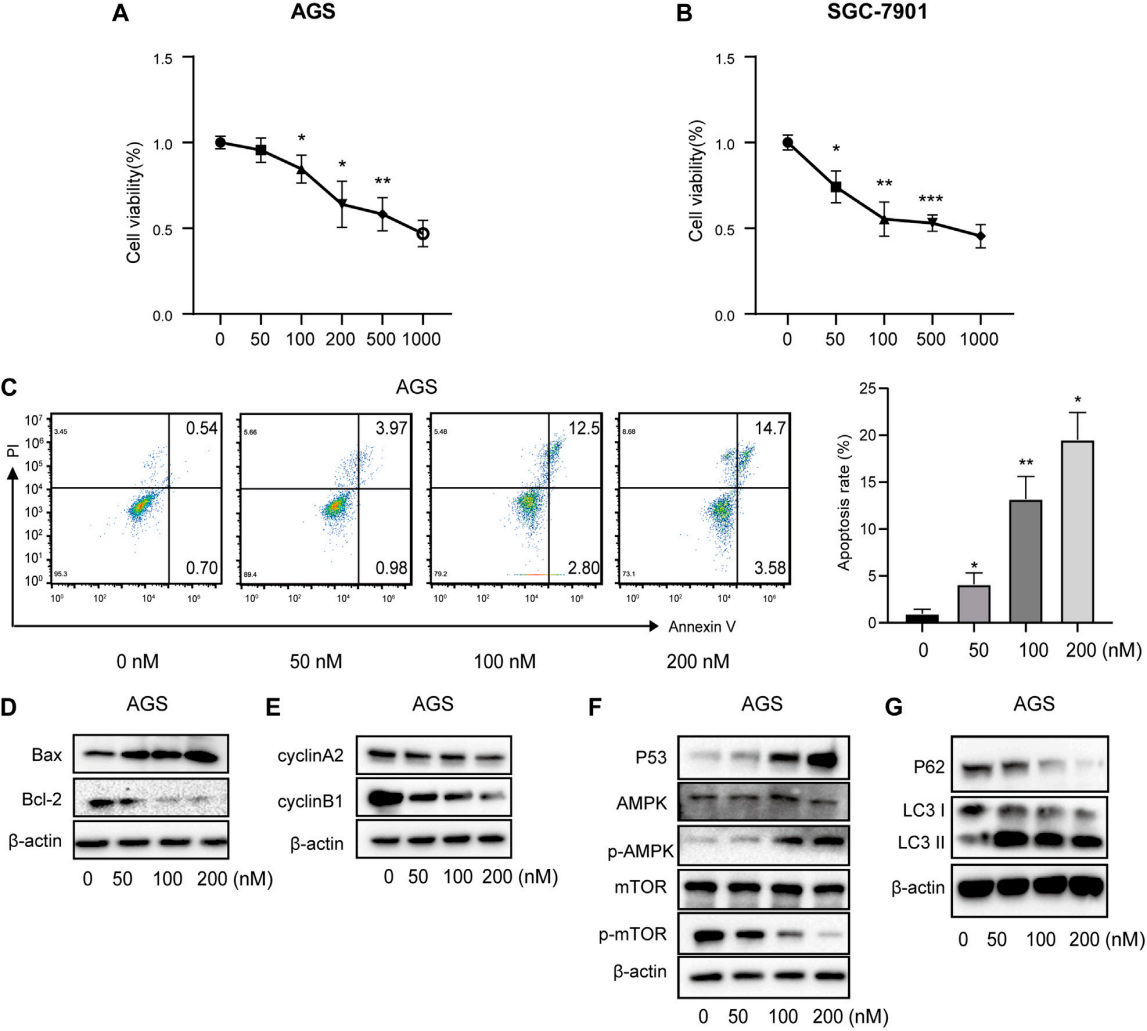
Vitamin D3 promotes apoptosis, autophagy, and inhibit cell cycle in AGS cells. **(A, B)**. Effect of vitamin D3 on AGS cells and SGC-7901 cells viability. **(C)**. The flow graph represents the pro-apoptotic effect of vitamin D3 on AGS cells. **(D–G)**. Detection of various protein expression in AGS cells by Western blotting.

Correlations between vitamin D3 and GC were analyzed by Network Pharmacology, and subsequently experimentally validated by assessing the mechanism underlying vitamin D3 inhibitory activity against AGS and SGC-7901 cells using the CCK-8 kit. The results indicated that vitamin D3 inhibited the proliferation of SGC-7901 and AGS cells (Figure 10A, B). These data confirm the antiproliferative effect of vitamin D3 in tumors. Based on these experiments, we selected suitable concentrations of vitamin D3 for use in subsequent experiments.

#### 3.3.2 Vitamin D3 promotes p53/AMPK/mTOR signaling and induces apoptosis of AGS and SGC-7901 cells

**FIGURE 11:**
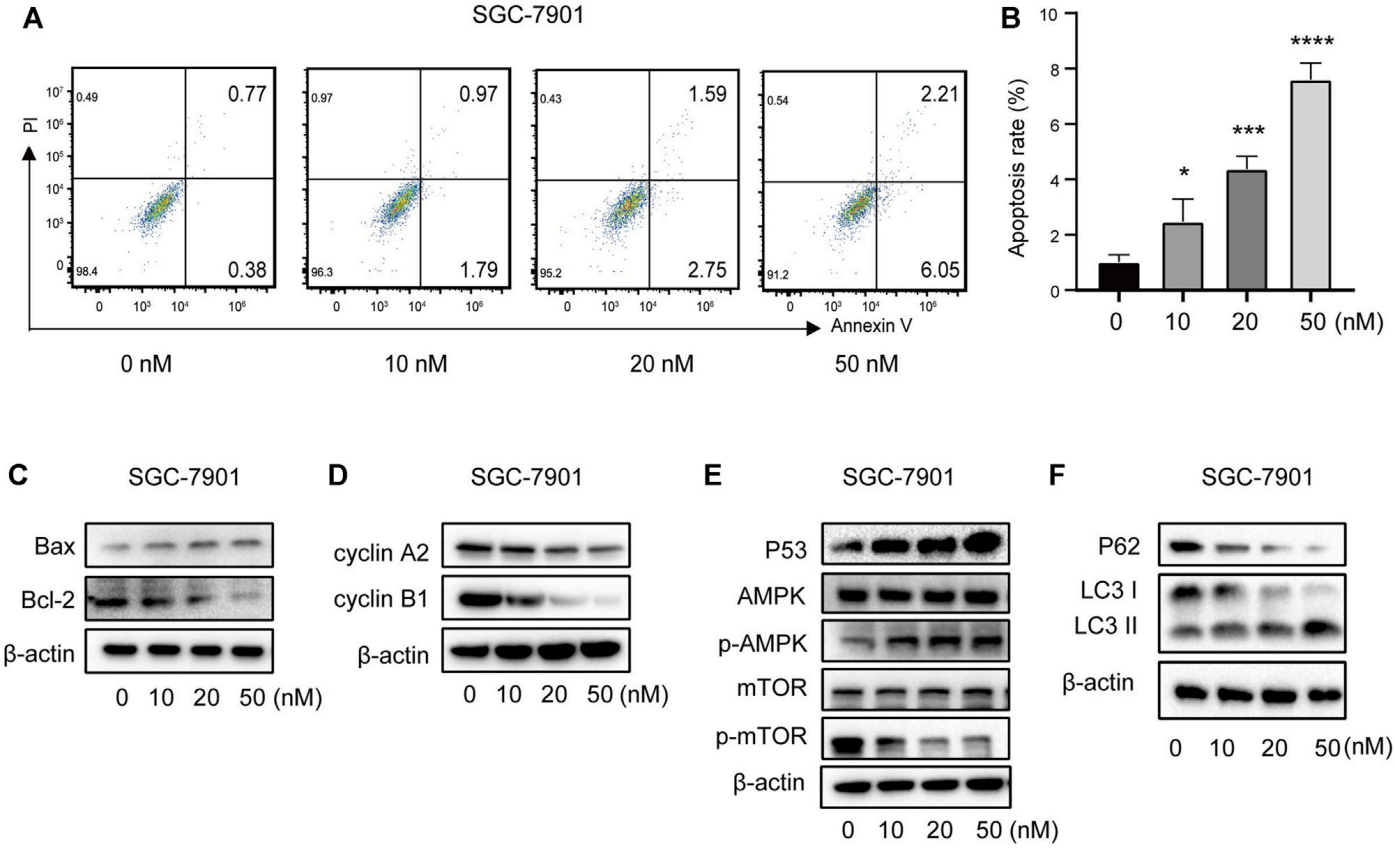
Vitamin D3 promotes apoptosis, autophagy, and inhibit cell cycle in SGC-7901 cells. **(A)** The flow graph represents the pro-apoptotic effect of vitamin D3 on SGC-7901 cells. **(B)** Bar graphs represent the apoptosis ratio of SGC-7901 cells at different concentrations. **(C–F)**. Detection of various protein expression in SGC-7901 cells by Western blotting.

To further verify the ability of vitamin D3 to inhibit GC cell proliferation, we assessed whether vitamin D3 promoted apoptosis of AGS and SGC-7901 cells, using Annexin V-FITC and PI staining. In the flow cytometry plot, our data indicated that the sum of early (the lower right quadrant) and late apoptosis (the upper right quadrant) increased with higher vitamin D3 concentration (Figure 10C; Figure 11A, B).

Further, we found that treatment of GC cells with vitamin D3 increased the production of the pro-apoptotic protein, Bax, and decreased that of the anti-apoptotic protein Bcl-2 (Figure 10D; Figure 11C). We also examined levels of the cell cycle proteins, cyclin A2 and cyclin B1, and found that both decreased after vitamin D3 treatment (Figure 10E; Figure 11D).

To determine whether vitamin D3 inhibits GC development through the p53/AMPK/mTOR pathway, we used Western blotting analysis to detect proteins in this pathway. In the vitamin D3 intervention group, no clear changes were found in the levels of total AMPK or mTOR proteins, whereas the levels of p53 and phospho-AMPK proteins were upregulated and that of phospho-mTOR was downregulated (Figure 10F; Figure 11E). Further, we found that P62 was downregulated and the LC3II/I ratio was upregulated (Figure 10G; Figure 11F), suggesting that vitamin D3 promoted autophagy and apoptosis, while suppressing proliferation through p53/AMPK/mTOR, p53/Bax/Bcl-2, p53/cyclin B1, and AMPK/cyclin A2 pathways in GC cell lines.

## 4 Discussion

Numerous signaling pathways have been identified as contributing to GC development in previous studies (Lei et al., 2022). In this investigation, the role of vitamin D3 in relation to GC was evaluated using network pharmacology techniques and possible molecular mechanisms underlying the role of vitamin D3 in GC predicted in a comprehensive analysis. Our network analysis findings were further verified using *in vitro* experiments.

Using network pharmacology analysis, we identified 60 drug-disease targets and screened 10 hub molecules. External verification analysis of the hub genes found that, except for *TPR* and *RNABP2*, mRNA levels of the other 8 genes were higher in GC tumors than in normal tissue. Further, the expression of CDK1, TOP2A, AURKB, CDCA8, CCNE1, and CDC25B were at higher levels in GC tumors, and high expression of RNABP2, CDC25B, CDC25A, CCNE1, and TPR was associated with poor patient prognosis. Various signaling pathways were identified as enriched for target genes by KEGG analysis. These findings were verified by *in vitro* cell experiments, which demonstrated that vitamin D3 upregulates the phosphorylation levels of AMPK, thus activating the AMPK pathway. Analysis of the AMPK pathway in the KEGG database showed that activation of the AMPK pathway can induce cell cycle arrest and autophagy. As our KEGG analysis demonstrated that cell cycle and apoptosis were enriched among the vitamin D3/GC targets identified in this study, we conducted further analyses of the cell cycle, apoptosis, and autophagy in this context.

Cell death is a physiological process that is necessary for homeostasis within organisms, but can sometimes lead to local or systemic inflammatory reactions, as well as organ dysfunction and disease (Chen et al., 2021). Cell death can be induced by various factors to prevent GC cell proliferation (Wang et al., 2022). Autophagy is a survival mechanism, but also plays a crucial role in cell death, distinct from that of apoptosis; autophagy and apoptosis are referred to as Type II and Type I programmed cell death, respectively (Kelekar, 2008; Teng et al., 2016). These two types of cell death share the same stimulatory factors and regulatory proteins, but have different thresholds for induction and how they are converted and coordinated remains unclear (Cao et al., 2021).

Autophagy, derived from the Greek roots “auto” and “phagy,” is a cellular self-digestion phenomenon, first proposed by De Duve in 1963 (Galluzzi et al., 2017), and regulated by autophagy-related genes (ATGs). The intracellular homeostatic process of autophagy is influenced by the proteins, light chain 3 (LC3), p62, and Beclin-1 (Schmitz et al., 2016; Zheng et al., 2017). When autophagy occurs, both autophagosome levels and LC3II/LC3I ratios increase (Gui et al., 2017), and may inhibit tumorigenesis by eliminating p62 (Mathew et al., 2009). Autophagy has recently become a focus of research into GC pathogenesis and targeted therapy (Kelekar, 2008; Teng et al., 2016), since GC can be either inhibited or promoted by autophagy (Cao et al., 2019). Further, abnormal autophagic activity can lead to degradation of cellular components necessary for GC cell maintenance and survival, ultimately leading to cell death and inhibition of tumor progression (Qian and Yang, 2016). Our experiments revealed an increase in the LC3II/LC3I ratio and a decrease in p62 protein expression with increasing concentrations of vitamin D3 in AGS and SGC-7901 cells. Further, vitamin D3 treatment induced GC cell autophagy, promoted cell death, and inhibited GC cell proliferation.

Both mTOR-dependent (e.g., the PI3K/Akt/mTOR and AMPK/mTOR pathways) and non-mTOR-dependent (e.g., the p53 pathway) signaling are associated with autophagy (Cao et al., 2019; Koustas et al., 2021; Rahman et al., 2021). Rapamycin mammalian target protein kinase (mTOR) has important roles in protein and lipid synthesis, cell proliferation, and other processes (Zhao et al., 2015). An increased AMP/ATP ratio promotes adenosine monophosphate-activated protein kinase (AMPK) activation of TSC1/TSC2 protein heterodimer inhibition of mTORC1 activity and induces autophagy (Zheng et al., 2015). AMPK also inhibits mTORC by directly phosphorylating Raptor, a protein component of mTORC1 (Gwinn et al., 2008). As a sequence-specific transcription factor, p53 promotes autophagy through the inducible factor, damage-regulated autophagy regulator 1 (DRAM1), and Sestrin2 (Zhou et al., 2016). Activation of p53 promotes AMPK activation, ultimately leading to GC cell autophagy (Feng et al., 2005; Hu et al., 2019; Gao et al., 2020). Previous studies have found that the MAPK, p53, HER2, and PI3K/AKT/mTOR signaling pathways are associated with GC development (Lei et al., 2022). AMPK/mTOR signaling also plays a role in GC development but has been the subject of fewer studies (Xiao et al., 2020). Our experiments demonstrated that treatment of GC tumor cells with vitamin D3 did not significantly influence total AMPK or mTOR levels, while p53 and p-AMPK protein expression rose, and p-mTOR expression gradually declined in a concentration-dependent manner. These results suggest that vitamin D3 can inhibit GC cell activity by inducing autophagy through the p53/AMPK/mTOR pathway.

Vitamin D3 can significantly activate apoptosis in undifferentiated malignant gastric cells (Ren et al., 2012), but the exact mechanism involved is unclear. We also found that target proteins were enriched in the apoptotic pathway by KEGG analysis (data not shown). Therefore, we conducted in depth analysis of the apoptotic pathway. Apoptosis can be triggered by two pathways: the exogenous pathway and the endogenous pathway. DNA damage and intracellular stimuli, such as hypoxia and oxidative stress, promote endogenous (mitochondrial) apoptosis. Pro-apoptotic (e.g., Bax) and anti-apoptotic (e.g., Bcl-2) proteins of the B-cell lymphoma 2 family interact to enhance mitochondrial membrane permeability (Singh et al., 2019). p53 is thought to regulate mitochondrial apoptosis through transcriptionally unrelated pathways (Aubrey et al., 2018). Further, p53 indirectly induces apoptosis by physically binding to the anti-apoptotic protein, Bcl-2 (Wei et al., 2021). Our data reveal that vitamin D3 can upregulate Bax expression and downregulate that of Bcl-2, as well as upregulating p53 expression in GC cells. Taken together, these findings suggest that vitamin D3 promotes GC cell apoptosis through p53/Bax/Bcl-2 pathway.

The biological processes of cell proliferation and the cell cycle interact (Eymin and Gazzeri, 2010). Previous studies have found that vitamin D causes G1 to S and S to G2 transition arrest in the GC cell cycle (Shah et al., 2021). The AMPK/cyclin A2 and p53/cyclin B1 pathways can block the tumor cell cycle, according to data in the KEGG database. Our experiments revealed that vitamin D3 inhibits cyclin A2 expression, which is predicted to result in GC cell cycle arrest in the transition from S to G2 phase. Cyclin B1 is expressed in G1, G2, and S phases, but its levels peak and its functions occur during M phase, and it is downregulated during the cell cycle transition to G2 after arrest, consistent with our experimental results. Therefore, our data demonstrate that vitamin D3 can prevent GC cell proliferation through cell cycle blockade via the p53/cyclin B1 and AMPK/cyclin A2 pathways.

In summary, our research demonstrates that vitamin D3 can facilitate cellular autophagy by activating the p53/AMPK/mTOR signaling pathway and inhibiting GC cell proliferation by meditating apoptosis and cell cycle arrest, thus providing a scientific basis for the effects of vitamin D3 in GC treatment. Our study has limitations, as our experiments were limited to *in vitro* cellular investigations, and more comprehensive and *in vivo* experiments are needed. For example, the molecular role of vitamin D3 treatment in GC could be further investigated by gene silencing, pathway inhibition, or activator methods. Cancer incidence and treatment outcomes may be improved by reducing vitamin D3 deficiency, which would be a safe and cost-effective approach.

In conclusion, we tested the molecular mechanism underlying the effects of vitamin D3 on GC using network pharmacology and *in vitro* experiments. Our data demonstrate that vitamin D3 can inhibit GC by up-regulating the p53/AMPK/mTOR signaling pathway, inducing autophagy, promoting apoptosis, and blocking the cell cycle, among other effects. We confirmed the reliability of our network pharmacology findings using experimental studies. Consequently, the molecular mechanisms elucidated in this investigation have strong scientific support.

## Data Availability

The original contributions presented in the study are included in the article/Supplementary Material, further inquiries can be directed to the corresponding authors.

## Supplementary material

SUPPLEMENTARY FIGURE S1 Molecular docking pattern of vitamin D3 with 5 hub target proteins. **(A)**. CDK1 **(B)**. TOP2A **(C)**. AURKB **(D)**. CDCA8 **(E)**. EZH2.

SUPPLEMENTARY FIGURE S2 The mRNA expression of 5 hub genes in tumor tissue (red) and normal tissue (grey) in the GEPIA database.

SUPPLEMENTARY FIGURE S3 The mRNA expression of 10 hub genes in different pathological stages.

SUPPLEMENTARY FIGURE S4 The protein expression of 5 hub genes in immunohistochemical images in the HPA database.

SUPPLEMENTARY FIGURE S5 Correlation between 5 hub genes and immune cell infiltration. **(A)** . CDK1 **(B)**. TOP2A **(C)**. AURKB **(D)**. CDCA8 **(E)**. EZH2.
