# Supplementary figures and images for: Vitamin D3 promotes gastric cancer cell autophagy by mediating p53/AMPK/mTOR signaling
Source: Front Pharmacol. 2024 Jan 8;14:1338260. doi: 10.3389/fphar.2023.1338260 (PMC10800859; doi:10.3389/fphar.2023.1338260)

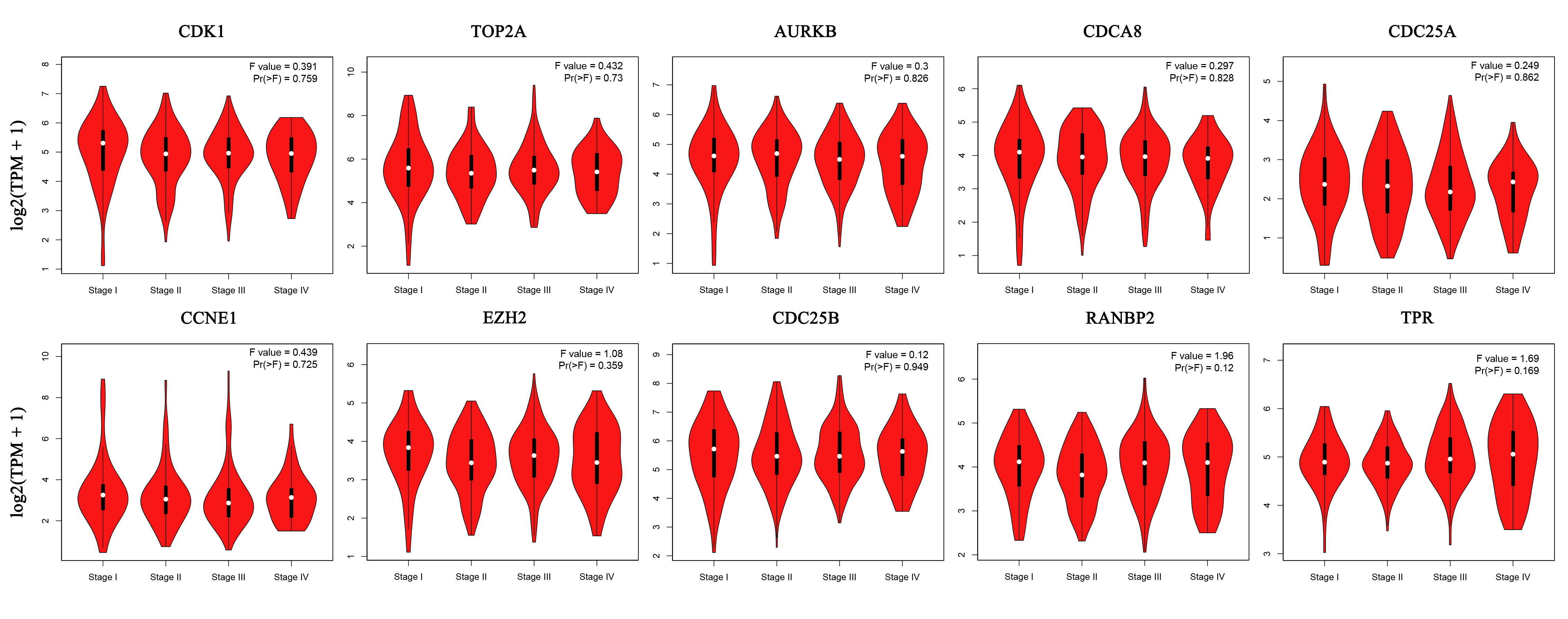

Supplement: Supplementary file 1 [file Image3.JPEG]

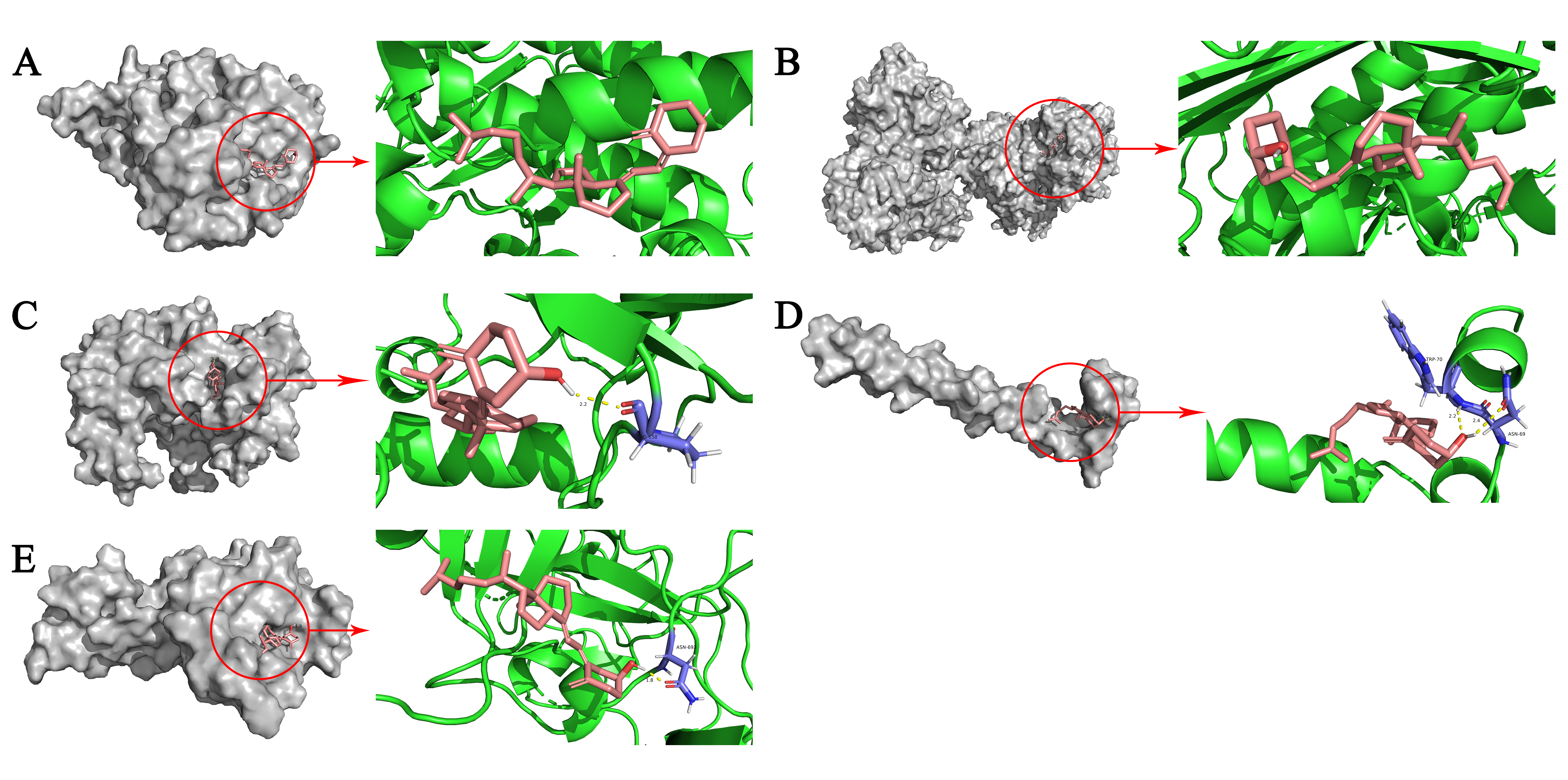

Supplement: Supplementary file 2 [file Image1.JPEG]

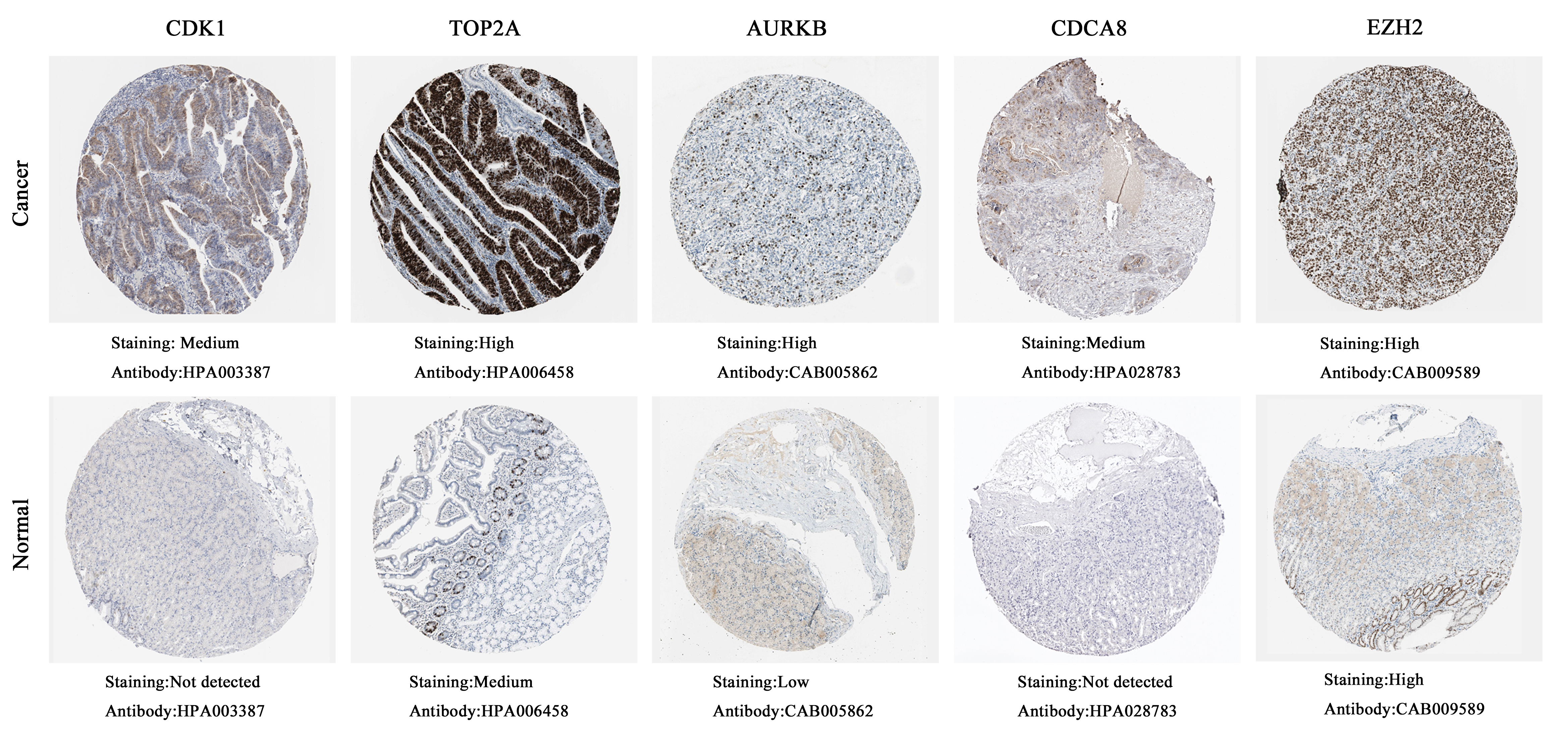

Supplement: Supplementary file 3 [file Image4.JPEG]

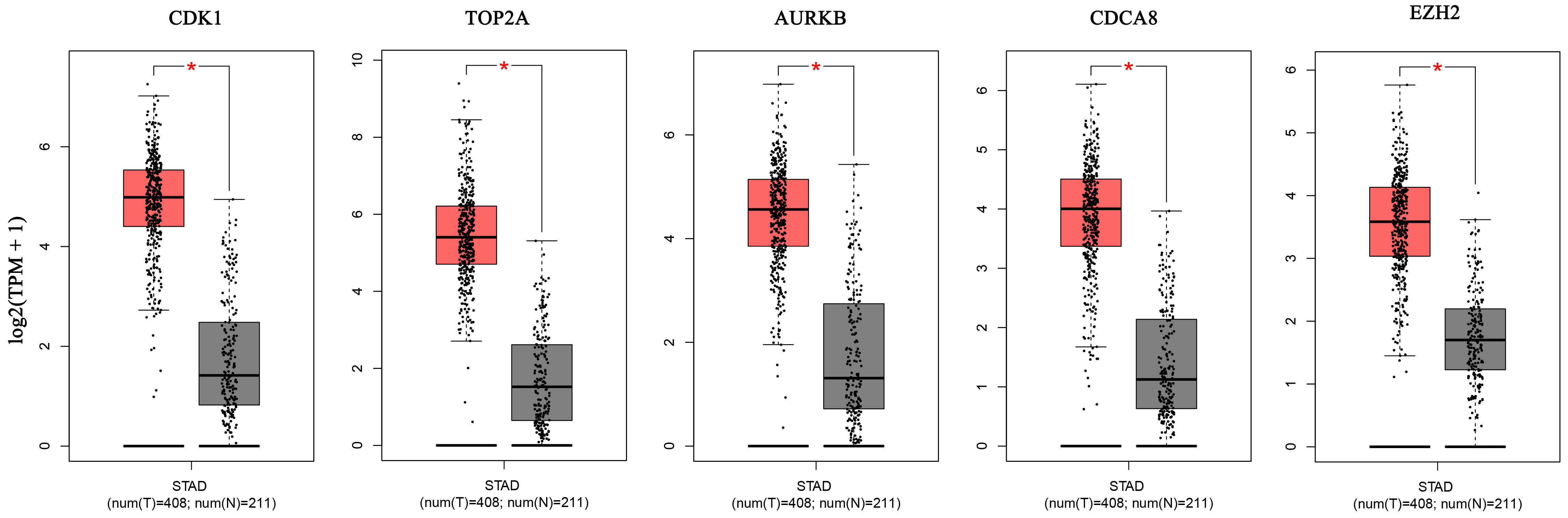

Supplement: Supplementary file 4 [file Image2.JPEG]

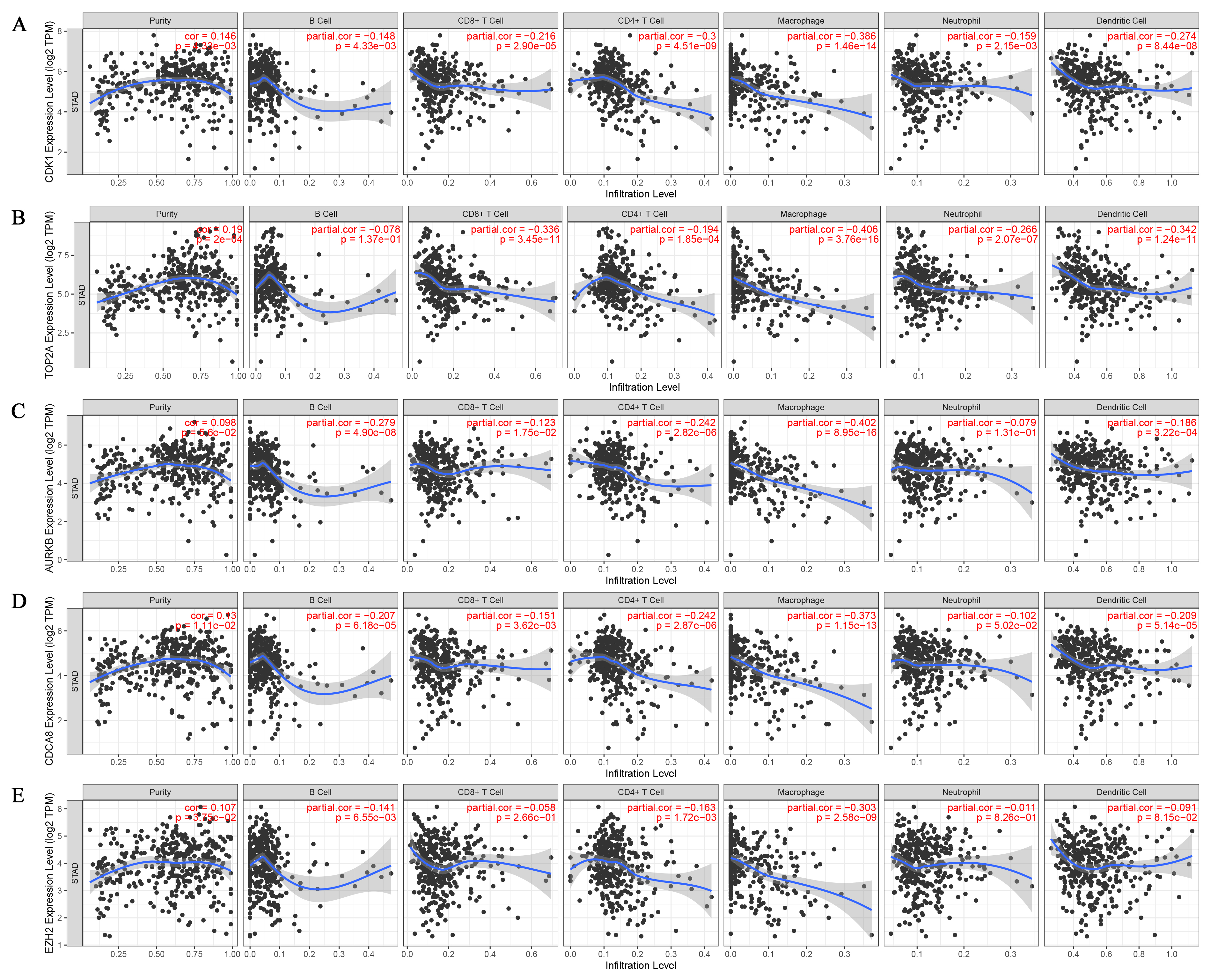

Supplement: Supplementary file 5 [file Image5.JPEG]
